# Demonstration of motion-compensated volumetric modulated arc radiotherapy on an MR-linac

**DOI:** 10.1016/j.phro.2025.100729

**Published:** 2025-02-15

**Authors:** Pim T.S. Borman, Prescilla Uijtewaal, Jeffrey Snyder, Bryan Allen, Caiden K. Atienza, Peter Woodhead, Daniel E. Hyer, Bas W. Raaymakers, Martin F. Fast

**Affiliations:** a Department of Radiotherapy, University Medical Center Utrecht, Utrecht, The Netherlands; b Department of Radiation Oncology, Yale New Haven Health, New Haven, CT, USA; c Department of Radiation Oncology, University of Iowa, Iowa City, IA, USA; d Elekta AB, Stockholm, Sweden

**Keywords:** MR-linac, VMAT, MLC tracking, Lung cancer

## Abstract

Intensity-modulated radiotherapy (IMRT) in combination with magnetic resonance imaging (MRI)-guided gated delivery represents the latest development in the treatment of abdominothoracic tumours on MR-linac. In contrast, volumetric-modulated arc therapy (VMAT) is typically used on conventional linacs due to its superior delivery efficiency and speed. Non-inferior VMAT plans were created in a research treatment planning system for eight lung cancer patients previously treated on an MR-linac. VMAT plans were delivered on a moving dosimeter using respiratory multi-leaf collimator (MLC) tracking. VMAT with MLC tracking achieved an average 2%/2 mm local gamma pass rate of 93% relative to planned dose with a delivery efficiency of 83%.

## 1 Introduction

Volumetric modulated arc therapy (VMAT) is considered the standard-of-care for radiotherapy on conventional C-arm linacs due to its ability to achieve highly conformal dose distribution in a time-efficient manner. MR-linacs are currently limited to step-and-shoot intensity-modulated radiotherapy (IMRT), but offer superior soft-tissue imaging that can be used for daily plan adaptation and real-time image-guided motion mitigation strategies [1]. Unfortunately, beam gating, which is standardly available on MR-linacs, can be challenging to combine with VMAT. The typically strict interdependence of gantry speed, leaf motion dynamics, and dose rate modulation during VMAT is easily disrupted by asynchronous gating events. Compatibility can, in principle, be achieved by reducing the gantry speed [2], at the risk of reducing part of the efficiency gain that VMAT offers compared to IMRT. Multi-leaf collimator (MLC) tracking, on the other hand, achieves dosimetric results that are highly comparable with gating while not disrupting the treatment delivery [3]. Previous studies demonstrated the compatibility of VMAT and MLC tracking on conventional linacs [4], [5] and MR-linacs [6]. In our previous MR-linac VMAT study [6], the dosimetry phantom was not physically moved, but required post-hoc motion-correction of the measured dose distributions. Additionally, the VMAT plans were not dosimetrically on par with IMRT, and the delivery system was not optimized for efficiency. In this study, the delivery system was improved by a better synchronization of MLC and linac controllers, enhanced stability at low gantry speeds, and improved handling of leaf pairs under the diaphragms. We present a technical demonstration of VMAT deliveries with MLC tracking on an MR-linac for lung cancer patients. Using a moving dosimeter, we demonstrate the high delivery efficiency of VMAT+tracking with respect to the IMRT+gating clinical baseline scenario while maintaining dosimetric non-inferiority.

## 2 Materials and methods

### 2.1 Patient selection

Eight lung cancer patients treated with gating on the 1.5 T Unity MR-linac (Elekta AB, Stockholm, Sweden) that consented to data sharing were included in this study. Their pathologies included lung adenocarcinoma, lung squamous cell carcinoma, small cell lung cancer, and leiomyosarcoma. The cancer stages at treatment ranged from I to IV. The majority of patients had disease adjacent to organs at high risk for radiation-induced toxicity, such as the heart, esophagus, trachea, and bronchus. The radiation dose per fraction was determined based on standard treatment regimens. For instance, a patient with a positive bronchial margin following a lobectomy received 1.8 Gy per fraction, while those with early-stage central lung cancers received higher doses per fraction [7], [8].

This research was reviewed and approved by the University of Iowa IRB-00000099 (Biomedical, application 201109821, Buatti principal investigator). The research was conducted in compliance with ICHE6(R2) as adopted by U.S. law. Prospective consent was obtained for all participants; none of the elements were waived. The research is considered exempt from Helsinki as it is not considered human subjects research as defined by the Declaration.

### 2.2 Treatment planning

Dual-arc MR-linac VMAT plans were created in Elekta’s research treatment planning system (TPS), Monaco v6.0.9, using a research leaf sequencer and dose optimization algorithm called OFL+PGD optimizer [9]. Dosimetric non-inferiority of Unity VMAT prostate plans as compared to the current step-and-shoot IMRT MR-linac plans has recently been reported [10]. Additionally, Unity VMAT prostate SBRT plans have been shown to meet standard dosimetric constraints as published through clinical trials such as PACE-B [11][12]. Similarly, in this study all VMAT lung plans met the same clinical target dose objectives and organ-at-risk dose constraints as the clinically delivered step-and-shoot IMRT plans.

### 2.3 Treatment delivery

**Fig. 1:**
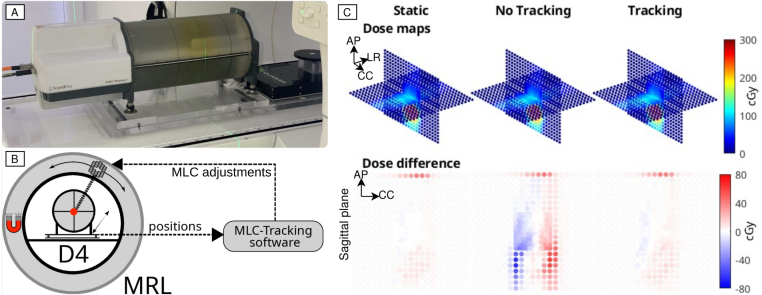
A: Phantom setup consisting of the Delta4 (D4) phantom placed on the Quasar motion MR platform. B: MLC tracking feedback loop. C: Example overview of the dose distributions of patient 2 measured with the Delta4.

All experiments were performed on a 1.5 T MR-linac in research mode, enabling VMAT in combination with MLC tracking [6]. The experimental setup is summarized in Fig. 1.

For motion-included dosimetry experimentation, the Delta4 Phantom+ MR (Scandidos AB, Uppsala, Sweden) was placed on a QUASAR Motion MR Platform (IBA Quasar, London ON, Canada) [13]. The centre of the Delta4 was positioned at isocenter in the sup–inf (SI) direction for all plans. In the left–right (LR) direction, two patient plans were measured at isocentre, three with an offset of −5cm, one with an offset of −8.5cm, and two with an offset of +6 cm. Different LR offsets were chosen to ensure that the high-dose area of the plan coincided with the high-density diode grid at the centre of the phantom. In the anterior–posterior (AP) direction, the central diode of the Delta4 was positioned at a fixed isocentre offset of +1.6 cm (lowest setting). All VMAT plans were recalculated on the Delta4 prior to performing dosimetric comparisons.

The motion platform was programmed to perform both artificial cos${}^{4}$ motion (20 mm peak–peak, 4 s period) as well as patient-derived motion in the SI direction. The patient-derived motion trace was based on the patient’s actual motion trace collected with the clinical *Comprehensive Motion Management* (CMM) software during the first IMRT fraction. The trace was smoothed with a median filter to remove the effects of position jitter and thereby accommodating the phantom’s speed limits.

In-house MLC tracking software with a closed-loop control system was implemented based on the stream of actual platform positions, see Fig. 1B, since the Delta4 cannot be imaged using MRI. The streamed platform positions were down-sampled to match the 6 Hz MRI acquisition frequency used in the Elekta’s motion monitoring software [14]. Down-sampled positions were used to build a linear regression prediction model that compensated for the 100 ms MLC latency, which was measured according to the procedure described by Uijtewaal et al. [15]. Predicted positions were then used to warp the MLC apertures according to the anticipated target position.

### 2.4 Evaluation

For each plan, dose deliveries with/without motion and with/without MLC tracking were compared to the planned dose by means of a local 2%/2 mm gamma scoring with a low-dose threshold of 20% of the maximum planned dose. A pass-rate of ≥90% was required to satisfy dosimetric accuracy constraints. Statistical significance was tested using a two-sided Wilcoxon signed rank test with a significance level of 0.05. The patient-recorded motion was characterized by the (95%–5% percentile) peak–peak distance and the dominant frequency. The delivery efficiency was calculated by dividing the actual average dose rate by the maximum dose rate (425 MU/min) of the MR-linac. The IMRT gating delivery time was retrospectively estimated from the TPS’s estimate. An assumed beam gating efficiency of 50% was used in conjunction with the assumption that the ungated delivery would consist of 50% move-only segments. Hence, the estimate from the TPS was multiplied by a factor 1.5.

## 3 Results

The gamma pass-rates and delivery statistics for all patients, motion traces, and delivery modes, are shown in Table 1. Note that the delivery time and efficiency are identical between tracking and non-tracking deliveries and are therefore listed only once. Exemplary dose distributions for patient 2 are shown in Fig. 1. These clearly show that large hot and cold spots appear relative to the planned dose without MLC tracking. MLC tracking of patient motion achieves excellent pass-rates which are above 90% in seven out of eight patients, and do not significantly differ from the static delivery. Gamma pass rates for MLC tracking deliveries were also significantly better than pass rates for non-tracking deliveries, with average increases of 37 (artificial motion) and 14 (patient-recorded motion) percentage points. The patient-recorded motion amplitude and period ranged from 3.3 mm to 11 mm peak–peak and from 2.8 s to 7.1 s, respectively. The mean absolute distance from zero correlated strongly (Pearson coefficient =0.96) with the gamma pass rates of the untracked delivery in the presence of patient motion. The delivery efficiency was high with an average of 83%, resulting in an average dose rate of 1.6 Gy per minute. The only noticeable outlier was patient 1 with an efficiency of 74%. This was likely caused by the low dose per arc, leading to higher average gantry and MLC speeds. Compared to the estimated delivery times of the gated IMRT reference plans, the median delivery times of the VMAT plans was 45% lower. In particular for patients 2 and 6 the improvement is striking: the IMRT+gating delivery time estimate is triple the VMAT+tracking delivery time.

**Table 1:** Dosimetric results including 2%/2 mm local gamma pass-rates delivery statistics. *None* refers to non-tracking while *track* denotes MLC tracking deliveries. The ${}^{*}$ symbol indicates a statistically significant difference (p < 0.05) relative to the static delivery, while the ${}^{†}$ symbol indicates a statistically significant difference (p < 0.05) between tracking and non-tracking. Note that the IMRT duration was estimated assuming a 50% gating duty cycle.

Pat.	Gamma pass rates					Delivery statistics				
	Static	$\cos^{4}$ motion		Pat. motion		Dose	Output	VMAT		IMRT
		none	track	none	track			Δt	Eff.	Δt
	[%]	[%]		[%]		[Gy]	[MU]	[min:sec]	[%]	[min:sec]
1	95.9	61.4	93.4	95.6	95.6	1.8	330	1:04	74	2:06
2	94.2	78.5	91.1	31.0	90.5	4.0	806	2:26	79	7:29
3	95.0	23.1	100.0	87.2	100	7.5	1515	4:15	85	8:51
4	89.4	54.4	80.6	73.7	85.4	8.0	2216	6:10	86	13:26
5	98.1	68.2	98.1	94.7	97.8	7.5	1393	3:59	83	5:14
6	95.6	41.9	88.2	83.2	90.4	7.5	1439	4:04	84	14:21
7	96.1	41.1	94.2	68.3	93.5	7.5	2044	5:31	88	9:06
8	99.6	70.0	99.1	99.1	98.7	7.5	2010	5:30	87	11:56

Avg.	95 ± 3	55 ± 18${}^{*}$	93 ± 6^†^	79 ± 22${}^{*}$	94 ± 5^†^	1.6 Gy or 350 MU/min			83 ± 5	

## 4 Discussion

In this phantom study, we demonstrated the time-efficient, dosimetrically accurate, and motion corrected delivery of clinical-grade VMAT plans for lung cancer patients on a 1.5 T MR-linac. The combined effect of dynamic segment motion of the VMAT delivery with additional (asynchronous) segment motion induced by MLC tracking did not lead to a significant degradation of dosimetric accuracy, or changes in delivery efficiency, relative to a static VMAT delivery without respiratory motion.

Our study demonstrates that MLC tracking is highly effective at mitigating respiratory motion while not suffering from the duty cycle penalty that is introduced by beam gating. Our VMAT plans displayed a high delivery efficiency of 83%, independent of the motion trace and therefore mainly determined by modulation complexity. This compares very favourable to the approximately 50% reached in step-and-shoot IMRT deliveries on the Unity MR-linac, using the clinical delivery system. Compared to our previous study by Uijtewaal et al. [6], we now benefited from the improved VMAT treatment planning capabilities of the OFL+PGD optimizer compared to the standard research Monaco that was previously used, and from the further optimized research delivery system, increasing deliverability and efficiency. In addition, the new motion platform, and CMM-recorded traces allowed us to directly measure pseudo-3D VMAT dose distributions subject to patient-specific motion, which was not possible in our previous studies.

Both MLC tracking and VMAT implementations on the 1.5 T MR-linac currently depend on research implementations of the treatment planning and delivery systems which makes them unsuitable for prospective clinical use. Given that all research functionality demonstrated in this study is embedded in clinical software and was designed with safety in mind, the road to future clinical implementation should be accessible.

Note that our dosimetric setup comes with several limitations. Because the Delta4 could not always be centred on the GTV due to the motion platform’s additional AP offset, the high dose area sometimes intersected with the peripheral detector region where the diode grid has a 10 mm spacing instead of 5 mm (Fig. 1). This most likely explains the reduced gamma pass-rates of some of the tracked cases compared to the static cases, most apparent for patient 4. When the tracked cases are directly compared to the static case, however, the pass-rates of this patient increased to 100% and 97.8%, for the patient and artificial motion respectively. In line with the current capabilities of the motion platform, respiratory motion remained limited to the SI direction in this study. While respiratory lung tumour motion is predominantly in SI, the addition of AP and LR motion would be desirable. Using an angulated phantom setup, we previously demonstrated that non-SI motion is not a limiting factor for MLC tracking, and can be efficiently compensated on the MR-linac using orthogonally moving jaws (Y collimators) and fast leaf speeds [6]. Another conceptual limitation of our experimental setup is that the Delta4 cannot be MRI scanned in line with vendor recommendations due to potential electronic damage. MLC tracking therefore had to be based on phantom reported positions which were down-sampled to match the typical MRI acquisition frequency. While this approach ignored image-induced motion estimation uncertainty, e.g. position jitter, such uncertainty would have a comparable impact on gating and MLC tracking deliveries. Additionally, the linear regression predictor used in this work is able to mitigate the additional latency caused by the imaging [6]. Another potential concern is degraded MRI accuracy due to continuous gantry rotation. For speeds up to 2 RPM the induced $B_{0}$ inhomogeneities are negligible [16], but for higher speeds this still needs to be verified. Despite these limitations, this work showed that clinically accepted VMAT plans can be delivered accurately and efficiently on the MR-linac, using MLC tracking for active motion managment.

## CRediT authorship contribution statement

**Pim T.S. Borman:** Conceptualization, Methodology, Supervision, Writing – review & editing, Investigation, Software, Formal analysis. **Prescilla Uijtewaal:** Formal analysis, Investigation. **Jeffrey Snyder:** Methodology, Writing – review & editing, Investigation. **Bryan Allen:** Investigation, Resources. **Caiden K. Atienza:** Formal analysis, Investigation. **Peter Woodhead:** Software, Writing – review & editing. **Daniel E. Hyer:** Methodology, Writing – review & editing, Investigation. **Bas W. Raaymakers:** Conceptualization, Writing review & editing. **Martin F. Fast:** Conceptualization, Funding acquisition, Methodology, Supervision, Writing – original draft, Writing – review & editing.

## Declaration of competing interest

The authors declare the following financial interests/personal relationships which may be considered as potential competing interests: The authors report research funding from Elekta AB. The authors report research agreements with IBA QUASAR. P. Woodhead is partially employed Elekta AB.
